# Let-7i-5p Mediates the Therapeutic Effects of Exosomes from Human Placenta Choriodecidual Membrane-Derived Mesenchymal Stem Cells on Mitigating Endotoxin-Induced Mortality and Liver Injury in High-Fat Diet-Induced Obese Mice

**DOI:** 10.3390/ph15010036

**Published:** 2021-12-27

**Authors:** Chao-Yuan Chang, Kung-Yen Chen, Hung-Jen Shih, Milton Chiang, I-Tao Huang, Yen-Hua Huang, Chun-Jen Huang

**Affiliations:** 1 Department of Medical Research, Wan Fang Hospital, Taipei Medical University, Taipei 116, Taiwan; yuanc669@gmail.com; 2 Graduate Institute of Clinical Medicine, College of Medicine, Taipei Medical University, Taipei 110, Taiwan; 3 Integrative Research Center for Critical Care, Wan Fang Hospital, Taipei Medical University, Taipei 116, Taiwan; 95352@w.tmu.edu.tw; 4 Department of Anesthesiology, Wan Fang Hospital, Taipei Medical University, Taipei 116, Taiwan; 5 Department of Anesthesiology, School of Medicine, College of Medicine, Taipei Medical University, Taipei 110, Taiwan; 6 Department of Surgery, Division of Urology, Changhua Christian Hospital, Changhua 500, Taiwan; jasta1206@gmail.com; 7 Department of Recreation and Holistic Wellness, MinDao University, Changhua 523, Taiwan; 8 Department of Urology, School of Medicine, College of Medicine, Taipei Medical University, Taipei 110, Taiwan; 9 International Master/Ph.D. Program in Medicine, College of Medicine, Taipei Medical University, Taipei 110, Taiwan; d142108016@tmu.edu.tw; 10 Emergency Department, Redcliffe Hospital, Brisbane, QLD 4020, Australia; itao.huang@uqconnect.edu.au; 11 School of Public Health, Faculty of Medicine, University of Queensland, Brisbane, QLD 4006, Australia; 12 Department of Biochemistry and Molecular Cell Biology, School of Medicine, College of Medicine, Taipei Medical University, Taipei 110, Taiwan; 13 TMU Research Center of Cell Therapy and Regeneration Medicine, Taipei Medical University, Taipei 110, Taiwan; 14 International Ph.D. Program for Cell Therapy and Regeneration Medicine, College of Medicine, Taipei Medical University, Taipei 110, Taiwan; 15 Center for Reproductive Medicine, Taipei Medical University Hospital, Taipei Medical University, Taipei 110, Taiwan

**Keywords:** obesity, sepsis, liver, exosomes, let-7i

## Abstract

Obesity complicates sepsis and increases the mortality of sepsis. We examined the effects of exosomes (from human placenta choriodecidual membrane-derived mesenchymal stem cells, pcMSCs) on preventing sepsis in obesity and the mitigating role of hsa-let-7i-5p microRNA. Obese mice (adult male C57BL/6J mice fed a high-fat diet for 12 weeks) received normal saline (HFD), endotoxin (10 mg/kg, intraperitoneal (ip); HFDLPS), endotoxin with exosomes (1 × 10^8^ particles/mouse, ip; HLE), or endotoxin with let-7i-5p microRNA inhibitor-pretreated exosomes (1 × 10^8^ particles/mouse, ip; HLEi). Our data demonstrated that the 48-h survival rate in the HLE (100%) group was significantly higher than in the HFDLPS (50%) and HLEi (58.3%) groups (both *p* < 0.05). In the surviving mice, by contrast, levels of liver injury (injury score, plasma aspartate transaminase and alanine transaminase concentrations, tissue water content, and leukocyte infiltration in liver tissues; all *p* < 0.05), inflammation (nuclear factor-κB activation, hypoxia-inducible factor-1α activation, macrophage activation, and concentrations of tumor necrosis factor-α, interleukin-6, and leptin in liver tissues; all *p* < 0.05), and oxidation (malondialdehyde in liver tissues, with *p* < 0.001) in the HLE group were significantly lower than in the HFDLPS group. Levels of mitochondrial injury/dysfunction and apoptosis in liver tissues in the HLE group were also significantly lower than in the HFDLPS group (all *p* < 0.05). Inhibition of let-7i-5p microRNA offset the effects of the exosomes, with most of the aforementioned measurements in the HLEi group being significantly higher than in the HLE group (all *p* < 0.05). In conclusion, exosomes mitigated endotoxin-induced mortality and liver injury in obese mice, and these effects were mediated by let-7i-5p microRNA.

## 1. Introduction

With over 650 million adults living with obesity worldwide, this condition is a global health concern [1]. Individuals with obesity are susceptible to multiple organ dysfunction and exhibit increased all-cause mortality [2]. As new adipocytes form and adipose tissue expands in individuals with obesity, an inadequate blood supply in the growing adipose tissues results in oxidative stress, which activates nuclear factor-κB (NF-κB) and induces inflammation [3]. Reduced oxygenation and subsequent oxidative stress further activate hypoxia-inducible factor-1α (HIF-1α), which enhances inflammation and polarizes macrophages to the proinflammatory M1 phase [4]. Because of this damage from oxidation and inflammation, mitochondrial dysfunction is frequently observed in obesity [5]; these mechanisms work synergistically to activate apoptosis and aggravate organ dysfunction [6]. These phenomena highlight the crucial roles of oxidation, inflammation, mitochondrial dysfunction, and apoptosis in mediating multiple organ dysfunction in obesity. However, effective therapies to prevent multiple organ dysfunction in patients with obesity are currently lacking [1,2].

Notably, obesity is associated with a higher risk of sepsis [7], which if untreated, causes multiple organ dysfunction and even mortality [8]. Oxidation, inflammation, mitochondrial dysfunction, and apoptosis are common pathogeneses of sepsis-induced multiple organ dysfunction [9]. For this reason, pathophysiological connections between obesity and sepsis should be highlighted; a reasonable assumption would be that obesity complicates sepsis and increases the mortality of sepsis. Although clinical data on this subject have been inconclusive [10,11], the notion was supported by data from a population-based study, which, during a 15-year follow-up, found that patients with obesity had a higher risk of mortality from bloodstream infection than did patients without obesity [12]. Because the prevalence of obesity is increasing in many populations [1], effective therapy to prevent sepsis in obesity is crucial.

Mesenchymal stem cells (MSCs) have a considerable antioxidation and anti-inflammation capacity as well as the ability to restore mitochondrial function [13]. Exosomes (nanosized extracellular vesicles [50–200 nm] with a lipid bilayer) have been identified as the key elements responsible for the therapeutic effects of MSC [14]. Moreover, exosome-based therapy has been reported to offer several advantages over MSC-based therapy [15]. For example, exosomes can produce superior therapeutic effects relative to MSC transplantation because exosomes are small and circulate readily, whereas MSC are too large to circulate and cannot move beyond the first-pass capillary bed, which is generally the lungs [15]. Moreover, the biological activity and functional properties of exosomes are more precise and, therefore, much easier to handle and produce in a scaled manner than those of MSC [15]. Notably, previous studies have indicated that microRNAs (miRNAs, a group of small noncoding RNAs) may be the active molecules responsible for mediating the effects of exosomes [16].

The present study thus investigated the therapeutic potential of exosomes (from human placenta choriodecidual membrane-derived MSCs, pcMSCs) to prevent sepsis in obesity. The hypothesis of this murine study was that exosome therapy would improve survival and alleviate sepsis-induced liver injury in obese mice. This study further investigated the modulating effects of exosomes on the crucial mechanisms involved. The possible role of miRNAs in producing these effects was also investigated in this murine study.

## 2. Results

### 2.1. Confirmation, miRNA Identification, and Biodistribution of Exosomes

**Figure 1:**
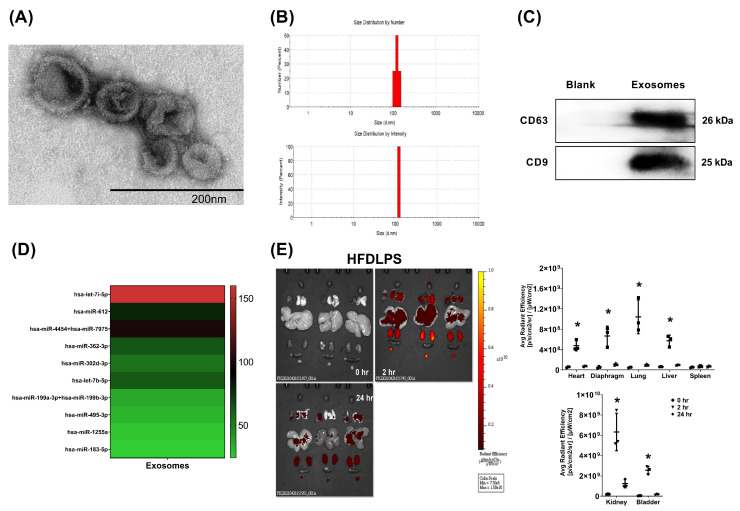
(**A**) Representative transmission electron microscopic images (60,000×) of exosomes (from human placenta choriodecidual membrane-derived mesenchymal stem cells, pcMSCs). (**B**) Particle and sizing analysis of exosomes. (**C**) Representative gel photography of exosome markers CD63 and CD9, detected using an immunoblotting assay. (**D**) The heatmap illustrating the top 10 most abundant microRNAs identified in exosomes. (**E**) Biodistribution of exosomes (conjugated with Cy7 mono NHS ester, 1 × 10^8^ particles per mouse) in endotoxin-treated high-fat diet obese (HFDLPS) mice measured at 0, 2, and 24 h after intraperitoneal administration, determined using an ex vivo bioluminescence imaging assay. Biodistribution data were derived from 3 mice at each time point and presented as the mean ± standard deviation. * *p* < 0.05, versus the signal intensity measured at 0 h.

The presence of exosomes was confirmed through the observation of a cup-shaped morphology, using transmission electron microscopy (TEM) (Figure 1A), a 100–150 nm particle size (Figure 1B), and by positive markers for CD63 and CD9, detected using an immunoblotting assay (Figure 1C). Hsa-let-7i-5p was the most abundant miRNA identified in the exosomes (Figure 1D). Bioluminescence imaging assay data revealed that in endotoxin-treated high-fat diet obese mice, at 2 h, but not at 24 h after administration, significantly higher signal intensities of Cy7-conjugated exosomes occurred in the heart, diaphragm, lungs, liver, kidney, and bladder (but not in the spleen) compared with at 0 h, (all *p* < 0.05; Figure 1E).

### 2.2. Survivorship, Metabolic Profiling, and Systemic Inflammation

**Figure 2:**
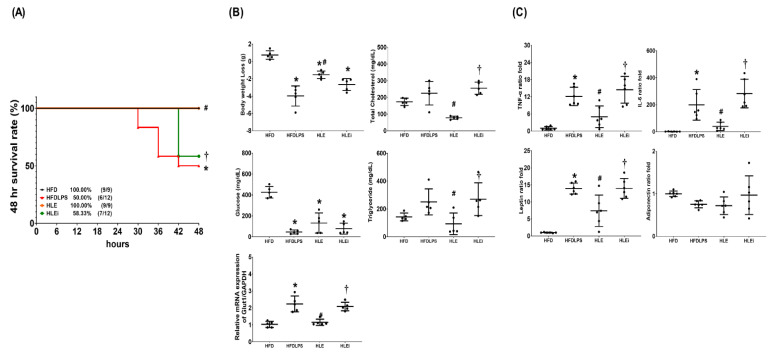
(**A**) The 48-h survival rates, as determined by calculating the number of mice in each group that survived the 48-h observational duration after endotoxin or normal saline (NS) administration. For this assay, 9, 12, 9, and 12 mice in each group were employed. (**B**) Body weight loss, concentrations of glucose, total cholesterol, and triglyceride in plasma, and the relative mRNA expression of glucose transporter 1 (GLUT1) in liver tissues (using a quantitative real-time polymerase chain reaction assay and normalized to the internal standard glyceraldehyde 3-phosphate dehydrogenase [GAPDH]), as measured at 48 h after endotoxin or NS administration in the surviving mice. Data were obtained from 5 mice in each group and presented as means ± standard deviations. (**C**) The ratio folds of tumor necrosis factor-α (TNF-α), interleukin-6 (IL-6), leptin, and adiponectin concentrations in plasma, as measured at 48 h after endotoxin or NS administration in the surviving mice using an enzyme-linked immunosorbent assay. Data were obtained from 5 mice in each group, compared to those of HFD group, and presented as means ± standard deviations. HFD: the high-fat diet plus intraperitoneal (ip) administration of NS (0.5 mL) group. HFDLPS: the high-fat diet plus endotoxin (10 mg/kg, ip) group. HLE: the high-fat diet plus endotoxin plus exosomes (from human placenta choriodecidual membrane-derived mesenchymal stem cells, pcMSCs, 1 × 10^8^ particles per mouse, ip) group. HLEi: the high-fat diet plus endotoxin plus microRNA-inhibitor-pretreated exosomes (1 × 10^8^ particles per mouse, ip) group. * *p* < 0.05, versus the HFD group; # *p* < 0.05, the HLE group versus the HFDLPS group. ^†^ *p* < 0.05, the HLEi group versus the HLE group.

Obese mice (adult male C57BL/6J mice fed a high-fat diet for 12 weeks) were randomly allocated to receive normal saline (HFD), endotoxin (10 mg/kg, intraperitoneal [ip]; HFDLPS), endotoxin with exosomes (1 × 10^8^ particles/mouse, ip; HLE), or endotoxin with let-7i-5p microRNA inhibitor-pretreated exosomes (1 × 10^8^ particles/mouse, ip; HLEi). Our data demonstrate that the 48-h survival rates in the HFD (100%) and HLE (100%) groups were comparable, and both of these rates were significantly higher than those of the HFDLPS (50%) and HLEi (58.3%) groups (all *p* < 0.05; Figure 2A).

Figure 2B presents the metabolic profiling and glucose transporter 1 (GLUT1) transcriptional expression data. Notably, significant decreases in body weight and plasma glucose concentrations were observed in mice in the HFDLPS, HLE, and HLEi groups. The magnitude of the body weight loss in the HFDLPS group was significantly greater than that in the HFD group (*p* < 0.001). Severe hypoglycemia was noted in the HFDLPS group, and the plasma glucose concentration in the HFDLPS group was significantly lower than that in the HFD group (*p* < 0.001). The magnitude of the body weight loss in the HLE group was significantly smaller than that in the HFDLPS group (*p* < 0.001). The plasma glucose concentration in the HLE group was higher than that in the HFDLPS group. However, the between-group difference in the plasma glucose concentration was nonsignificant (*p* = 0.170). Moreover, the magnitude of the body weight loss and plasma glucose concentrations in the HLE and HLEi groups were not significantly different. The total cholesterol and triglyceride concentrations in plasma in the HFDLPS group did not differ significantly from those in the HFD group (*p* = 0.241 and 0.230, respectively). By contrast, the total cholesterol and triglyceride concentrations in plasma in the HLE group were significantly lower than those in the HFDLPS (*p* < 0.001 and = 0.046, respectively) and HLEi (*p* < 0.001 and =0.023, respectively) groups. The mRNA levels of GLUT1 in livers in the HFDLPS group were significantly higher than those in the HFD group (*p* < 0.001). By contrast, the expression levels of GLUT1 in the HLE group were significantly lower than those in the HFDLPS and HLEi groups (both *p* < 0.001).

Figure 2C displays the data for the plasma concentrations of TNF-α, IL-6, leptin, and adiponectin. Comparisons of the plasma concentrations of TNF-α, IL-6, and leptin between groups yielded the same results as those for GLUT1. However, the plasma adiponectin concentrations in the HFD, HFDLPS, HLE, and HLEi groups were not significantly different.

### 2.3. Liver Injury and Oxidation

**Figure 3:**
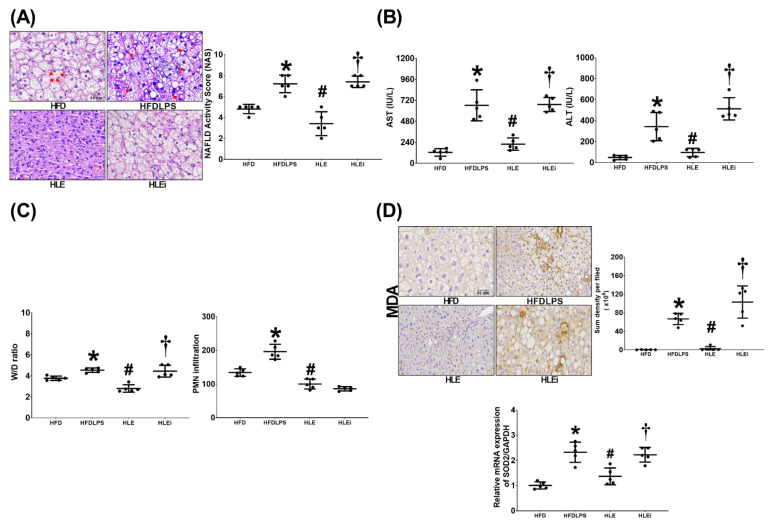
(**A**) Representative microscopic findings of the liver tissues stained with hematoxylin-eosin (200 ×) and the organ injury scores (NAFLD activity scores). Data were obtained from 5 mice in each group. (**B**) Plasma concentrations of hepatic enzymes aspartate aminotransferase (AST) and alanine aminotransferase (ALT). Data were obtained from 5 mice in each group (**C**). Wet/dry weight ratio (W/D ratio, an indicator of tissue water content) and polymorphonuclear leukocyte (PMN) infiltration levels in liver tissues. Data were obtained from 5 mice in each group (**D**). Representative microscopic images of malondialdehyde (MDA, an indicator of oxidation, using an immunohistochemistry staining assay) and the quantitative sum intensities of MDA in liver tissues. The relative mRNA expression of superoxide dismutase 2 (SOD2) in liver tissues (using a quantitative real-time polymerase chain reaction assay and normalized to the internal standard glyceraldehyde 3-phosphate dehydrogenase [GAPDH]). Data were obtained from 5 mice in each group. All assays were performed 48 h after endotoxin or normal saline (NS) administration in the surviving mice. Data were presented as means ± standard deviations. HFD: the high-fat diet plus intraperitoneal (ip) administration of NS (0.5 mL) group. HFDLPS: the high-fat diet plus endotoxin (10 mg/kg, ip) group. HLE: the high-fat diet plus endotoxin plus exosomes (from human placenta choriodecidual membrane-derived mesenchymal stem cells, pcMSCs, 1 × 10^8^ particles per mouse, ip) group. HLEi: the high-fat diet plus endotoxin plus microRNA-inhibitor-pretreated exosomes (1 × 10^8^ particles per mouse, ip) group. NAFLD: non-alcoholic fatty liver disease. * *p* < 0.05, versus the HFD group; # *p* < 0.05, the HLE group versus the HFDLPS group. ^†^ *p* < 0.05, the HLEi group versus the HLE group.

Figure 3A presents the histological characteristics and injury scores of the groups. We observed significant liver injury characteristics in the HFD, HFDLPS, HLE, and HLEi groups. The injury score for the HFDLPS group was significantly higher than that for the HFD group (*p* < 0.001). By contrast, the injury score for the HLE group was significantly lower than that for the HFDLPS and HLEi groups (both *p* < 0.001).

Comparisons of the plasma aspartate aminotransferase (AST) and alanine aminotransferase (ALT) data (Figure 3B) and the wet/dry weight (W/D) ratio and polymorphonuclear leukocyte infiltration data (Figure 3C) between groups yielded the same results as those for the injury scores data (except for the levels of polymorphonuclear leukocyte infiltration in the liver tissues of the HLE and HLEi groups not being significantly different [*p* = 0.453]). Liver oxidation was determined by assaying the expression of malondialdehyde and superoxide dismutase 2 (SOD2) in liver tissues. As illustrated in Figure 3D, comparison results of the malondialdehyde and SOD2 data between groups also paralleled those of the injury scores data.

### 2.4. Liver Inflammation

**Figure 4:**
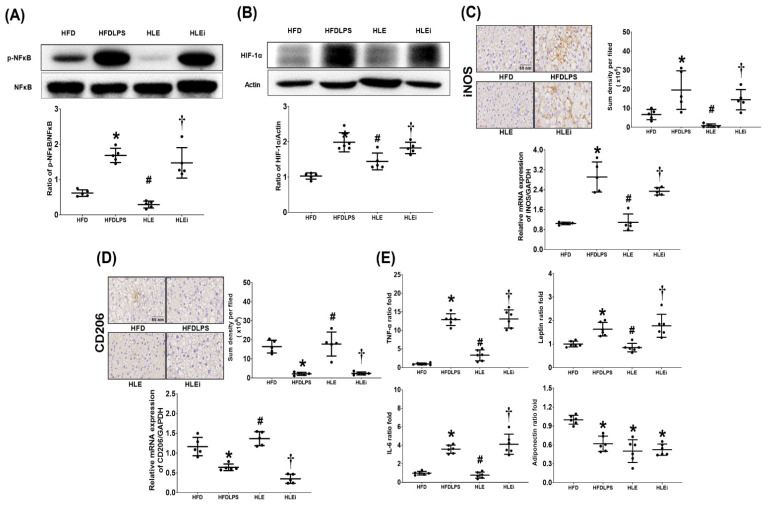
(**A**) Representative gel photographs of nuclear factor-κB (NF-κB) and phosphorylated NF-κB (p-NF-κB) in liver tissues analyzed using an immunoblotting assay and the relative band density of p-NF-κB/NF-κB in liver tissues, using an immunoblotting assay. Data were obtained from 5 mice in each group. (**B**) Representative gel photographs of hypoxia-inducible factor-1α (HIF-1α) and actin (the internal standard) in liver tissues analyzed using an immunoblotting assay and the relative band density of HIF-1α/actin in liver tissues, using an immunoblotting assay. Data were obtained from 5 mice in each group. (**C**) Representative microscopic images of inducible nitric oxide synthase (iNOS, the indicator of pro-inflammatory M1 phase macrophage polarization, using an immunohistochemistry staining assay) and the quantitative sum intensities of iNOS in liver tissues. The relative mRNA expression of iNOS in liver tissues (using a quantitative real-time polymerase chain reaction assay and normalized to the internal standard glyceraldehyde 3-phosphate dehydrogenase [GAPDH]). Data were obtained from 5 mice in each group. (**D**) Representative microscopic images of CD206 (the indicator of anti-inflammatory M2 phase macrophage polarization, detected using an immunohistochemistry staining assay) and the quantitative sum intensities of CD206 in liver tissues. The relative mRNA expression of CD206 in liver tissues (using a quantitative real-time polymerase chain reaction assay and normalized to GAPDH) in liver tissues. Data were obtained from 5 mice in each group. (**E**) The ratio folds of tumor necrosis factor-α (TNF-α), interleukin-6 (IL-6), leptin, and adiponectin concentrations in liver tissues, analyzed using enzyme-linked immunosorbent assays. The TNF-α, IL-6, leptin, and adiponectin data from each group were compared to that of the HFD group to determine the ratio fold. Data were obtained from 6 mice in each group. All assays were performed 48 h after endotoxin or normal saline (NS) administration in the surviving mice. Data were presented as means ± standard deviations. HFD: the high-fat diet plus intraperitoneal (ip) administration of NS (0.5 mL) group. HFDLPS: the high-fat diet plus endotoxin (10 mg/kg, ip) group. HLE: the high-fat diet plus endotoxin plus exosomes (from human placenta choriodecidual membrane-derived mesenchymal stem cells, pcMSCs, 1 × 10^8^ particles per mouse, ip) group. HLEi: the high-fat diet plus endotoxin plus microRNA-inhibitor-pretreated exosomes (1 × 10^8^ particles per mouse, ip) group. * *p* < 0.05, versus the HFD group; # *p* < 0.05, the HLE group versus the HFDLPS group. ^†^ *p* < 0.05, the HLEi group versus the HLE group.

Liver inflammation was determined by assaying the levels of nuclear factor-κB (NF-κB) and hypoxia-inducible factor-1α (HIF-1α) activation, macrophage activation (inducible nitric oxide synthase [iNOS] and CD206 expression), and concentrations of tumor necrosis factor-α (TNF-α), interleukin-6 (IL-6), leptin, and adiponectin in the liver tissues of HFD mice. The NF-κB data are presented in Figure 4A. The expression levels of phosphorylated-NF-κB in the HFDLPS group were significantly higher than those in the HFD group (*p* < 0.001). By contrast, the expression levels of phosphorylated-NF-κB in the HLE group were significantly lower than those in the HFD and HLEi groups (both *p* < 0.001). Notably, comparison results of the HIF-1α (Figure 4B), iNOS (Figure 4C), TNF-α, IL-6, and leptin data (Figure 4E) between groups paralleled those of the phosphorylated-NF-κB data. Notably, the immunohistochemistry assay data demonstrated that the expression levels of CD206 in the HFDLPS group were significantly lower than those in the HFD group (*p* < 0.001). By contrast, the expression levels of CD206 in the HLE group were significantly higher than those in the HFDLPS and HLEi groups (both *p* < 0.001). The CD206 real-time PCR data paralleled the immunohistochemistry assay data. Moreover, the adiponectin concentration in livers in the HFDLPS group was significantly lower than that in the HFD group (*p* < 0.001; Figure 4E). However, the adiponectin concentrations in livers in the HFDLPS, HLE, and HLEi groups did not differ significantly.

### 2.5. Mitochondrial Injury and Dysfunction

**Figure 5:**
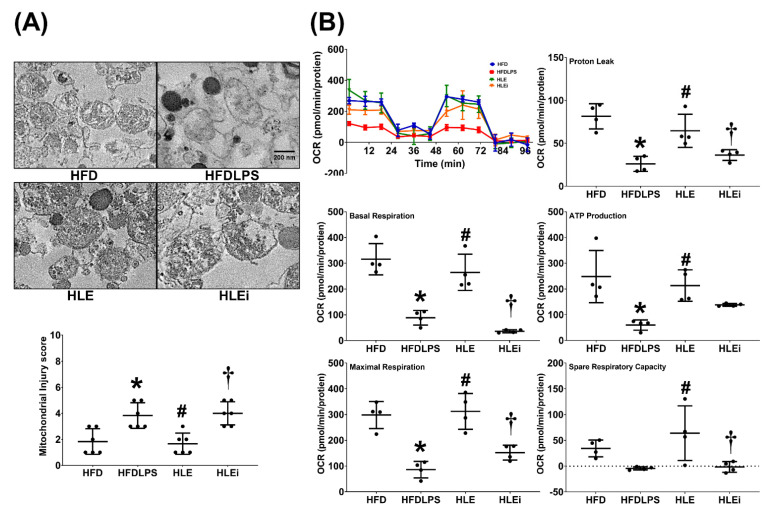
(**A**) Representative transmission electron microscope images of mitochondria morphology (8000×) and mitochondrial injury scores in primary hepatocytes isolated from liver tissues of high-fat diet obese mice. For analysis, 6 mice from each group and 4 random fields per grid (2 μm^2^) were selected. (**B**) Representative results of the mitochondrial respiratory function of hepatocytes, determined using a Seahorse Analyzer. Data were derived from 4 mice in each group. All assays were performed 48 h after endotoxin or phosphate buffered saline (PBS) administration in the surviving mice. Data were presented as means ± standard deviations. OCR, oxygen consumption rate. ATP: adenosine triphosphate. HFD: the high-fat diet plus PBS (100 μL) group. HFDLPS: the high-fat diet plus endotoxin (100 ng/mL) group. HLE: the high-fat diet plus endotoxin plus exosomes (from human placenta choriodecidual membrane-derived mesenchymal stem cells, pcMSCs, 1 × 10^9^ particles) group. HLEi: the high-fat diet plus endotoxin plus microRNA-inhibitor-pretreated exosomes (1 × 10^9^ particles) group. * *p* < 0.05, versus the HFD group; # *p* < 0.05, the HLE group versus the HFDLPS group. ^†^ *p* < 0.05, the HLEi group versus the HLE group.

Levels of mitochondrial injury and dysfunction were determined by assaying alterations in mitochondrial ultrastructure and mitochondrial respiratory function in primary hepatocytes isolated from the liver tissue of HFD mice. The mitochondrial injury data are presented in Figure 5A. We observed significant characteristics of mitochondrial injury in the HFD, HFDLPS, HLE, and HLEi groups. The mitochondrial injury score for the HFDLPS group was significantly higher than that for the HFD group (*p* = 0.006). By contrast, the mitochondrial injury score for the HLE group was significantly lower than that for the HFDLPS and HLEi groups (*p* = 0.003 and 0.002, respectively). The mitochondrial respiratory function data are displayed in Figure 5B. Basal respiration in the HFDLPS group was significantly lower than that in the HFD group (*p* < 0.001). By contrast, basal respiration in the HLE group was significantly higher than that in the HFDLPS and HLEi groups (*p* = 0.001 and < 0.001, respectively). Notably, comparison results of the maximal respiration, proton leak, ATP production, and spare respiratory capacity data between groups paralleled those of the basal respiration data. However, the difference in adenosine triphosphate (ATP) production between the HLE and HLEi groups (*p* = 0.335) and the difference in spare respiratory capacity between the HFD and HFDLPS groups (*p* = 0.259) were nonsignificant.

### 2.6. Apoptosis in the Liver

**Figure 6:**
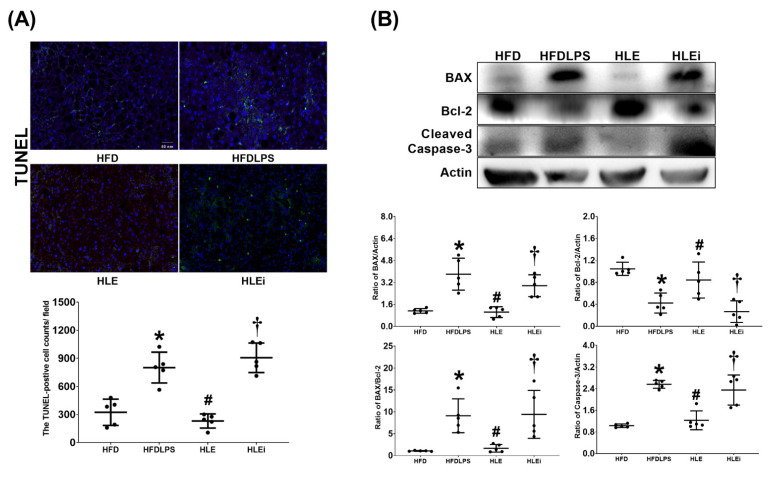
(**A**) Representative DNA fragmentation microscopic images (the indicator of apoptosis, marked by green fluorescence dots, 200×) in liver tissues assayed using the terminal deoxynucleotidyl transferase dUTP nick end labeling (TUNEL) method and the count of TUNEL-positive cells (0.25 mm^2^). Data were obtained from 5 mice in each group (**B**) Representative gel images of the proapoptotic BAX, the antiapoptotic Bcl-2, proapoptotic cleaved caspase-3, and actin (the internal standard) in liver tissues assayed using an immunoblotting assay, and the relative band density of BAX/actin, Bcl-2/actin, cleaved caspase-3/actin, and BAX/Bcl-2 ratios in liver tissues. Data were obtained from 5 mice in each group. All assays were performed 48 h after endotoxin or normal saline (NS) administration in the surviving mice. Data were presented as means ± standard deviations. HFD: the high-fat diet plus intraperitoneal (ip) administration of NS (0.5 mL) group. HFDLPS: the high-fat diet plus endotoxin (10 mg/kg, ip) group. HLE: the high-fat diet plus endotoxin plus exosomes (from human placenta choriodecidual membrane-derived mesenchymal stem cells, pcMSCs, 1 × 10^8^ particles per mouse, ip) group. HLEi: the high-fat diet plus endotoxin plus microRNA-inhibitor-pretreated exosomes (1 × 10^8^ particles per mouse, ip) group. * *p* < 0.05, versus the HFD group; # *p* < 0.05, the HLE group versus the HFDLPS group. ^†^ *p* < 0.05, the HLEi group versus the HLE group.

The presence of liver apoptosis was determined by assaying DNA fragmentation (through the terminal deoxynucleotidyl transferase dUTP nick end labeling [TUNEL] method); the expression of proapoptotic BAX, antiapoptotic Bcl-2, and proapoptotic cleaved caspase-3; and the BAX/Bcl-2 ratio in liver tissues. The TUNEL assay data are presented in Figure 6A. The TUNEL-positive cell counts in the HFDLPS group were significantly higher than those in the HFD group (*p* < 0.001). By contrast, the TUNEL-positive cell counts in the HLE group were significantly lower than those in the HFDLPS and HLEi groups (both *p* < 0.001). The BAX, Bcl-2, BAX/Bcl-2 ratio, and cleaved caspase-3 data are displayed in Figure 6B. Notably, comparison results of the BAX, BAX/Bcl-2 ratio, and cleaved caspase-3 data between groups paralleled those of the TUNEL-positive cell count data. By contrast, the expression levels of Bcl-2 in the HFDLPS group were significantly lower than those in the HFD group (*p* = 0.002). Moreover, the expression levels of Bcl-2 in the HLE group were significantly higher than those in the HFDLPS and the HLEi groups (*p* = 0.038 and 0.004, respectively).

## 3. Discussion

Data from this study demonstrate that exosomes can improve survivorship and mitigate metabolic alterations, systemic inflammation, and liver injury in endotoxin-treated obese mice. Our data also demonstrate that exosomes can mitigate oxidation, inflammation, mitochondrial injury and dysfunction, and apoptosis in the liver tissues of endotoxin-treated obese mice. Collectively, these data supported our hypothesis and demonstrated the therapeutic effectiveness of exosomes in preventing sepsis in obesity. Because the global population of adults with obesity is increasing and effective therapy for the prevention of sepsis in obesity is currently lacking [1], the data from the present study have profound clinical implications.

Previous data have indicated that, by upregulating IL-6, endotoxins can cause significant body weight loss in obese mice [17]. Similar results were observed in this study; our data revealed that endotoxins cause significant systemic inflammation (upregulation of TNF-α, IL-6, and leptin in plasma) and metabolic alterations (body weight loss and severe hypoglycemia) in obese mice. We also observed that endotoxins can cause liver injury and induce oxidation, inflammation (NF-κB and HIF-1α activation; upregulation of TNF-α, IL-6, and leptin; and M1 but not M2 phase macrophage polarization), mitochondrial injury and dysfunction, and apoptosis in liver tissues in obese mice. These data are consistent with previous endotoxemia data, which revealed that mitochondrial dysfunction may reduce noncarbohydrate precursor use and glucose production and eventually cause hypoglycemia [18]. Notably, a correlation between systemic inflammation, hypoglycemia, and mortality in patients with severe sepsis has been reported [19,20]. Considering the results of these reports [17,18,19,20], our observation of significant mortality in endotoxin-treated obese mice in this study is reasonable. Moreover, because exosomes can mitigate systemic inflammation and somewhat alleviate severe hypoglycemia, our observation that exosomes improved survivorship in endotoxin-treated obese mice was also reasonable.

Previous data have demonstrated the benefits of exosome therapy (for example, with exosomes from human umbilical cord MSC and adipose-derived MSC) in obese mice, including in restoring metabolic homeostasis, reducing oxidation and inflammation, and polarizing macrophages toward an anti-inflammatory M2 phase [21,22]. The preventive effects of exosomes from bone marrow MSC against sepsis, including mitigating acute lung injury and inhibiting HIF-1α in mice with endotoxemia, have also been reported [23]. Data from the present study clearly demonstrate that exosomes from human placenta-derived MSC can produce similar preventive effects against sepsis in obesity. Oxidative stress is a strong activator of NF-κB and HIF-1α, and the instrumental roles of NF-κB and HIF-1α in inducing inflammation have been well established [3,4]. Moreover, oxidation and inflammation may work in concert in causing mitochondrial injury and dysfunction [5]. Oxidation, inflammation, and mitochondrial dysfunction may further work synergistically to induce apoptosis [6]. Because exosomes from MSC can mitigate oxidation, inhibit NF-κB and HIF-1α, and suppress inflammation, our observations of the effects of exosomes from human placenta-derived MSC in mitigating mitochondrial dysfunction and apoptosis in the liver tissues of endotoxin-treated obese mice are also reasonable. Collectively, these data revealed that exosomes from human placenta-derived MSC may mitigate liver injury in endotoxin-treated obese mice by mitigating oxidation, inflammation, mitochondrial injury and dysfunction, and apoptosis.

Through base-pairing with the 3′-untranslated region of target mRNAs, miRNAs can regulate gene expression at the post-transcriptional level [24]. Notably, let-7i-5p miRNA was identified as the most abundant miRNA in exosomes from human placenta-derived MSC in this study. Previous data have revealed the therapeutic potential of let-7i-5p miRNA, as its overexpression could suppress hypoxia-induced NF-κB activation, mitochondrial dysfunction, and apoptosis in cardiomyocytes [25]. The therapeutic potential of let-7i-5p miRNA was also observed in this study; our data revealed that inhibition of let-7i-5p miRNA significantly offset the therapeutic effects of exosomes from human placenta-derived MSC in preventing sepsis in obesity. On the basis of these data, we propose that the therapeutic effects of exosomes from human placenta-derived MSC in preventing sepsis in obesity, are partially mediated by let-7i-5p miRNA. In line with this notion, we further conjecture that overexpressing let-7i-5p miRNA may very likely enhance the therapeutic potential and strengthen the therapeutic effects of exosomes from human placenta-derived MSC against sepsis in obesity. To investigate further on this issue, a follow-up murine study with the construction of human placenta-derived MSC that can overexpress let-7i-5p miRNA, through lipofectamine-mediated transfection of human placenta-derived MSC with let-7i-5p miRNA expression clones, is currently being conducted in our laboratory. Clinical data have indicated that patients with obesity may have superior outcomes to patients with average weights during sepsis, and the possibility of an “obesity paradox” was thus suggested [11]. However, this concept has been challenged; previous data derived from a study of 64,027 individuals with a 15-year follow-up revealed a higher mortality in obese patients with sepsis compared to that in patients without obesity [12]. This study employed gram-negative endotoxins to induce monomicrobial sepsis [26]. The dose of endotoxin used in this study was lower than that of our previous study (10 vs. 15 mg/kg) [27]. The reason for this was that our preliminary data revealed a higher mortality rate in obese mice treated with 15 mg/kg endotoxin than in nonobese mice (100% vs. 85.7%) over a 48-h observation period [27]. These data, in concert with those of previous studies [10,12], support the notion that obesity complicates sepsis and increases the mortality of sepsis.

**Figure 7:**
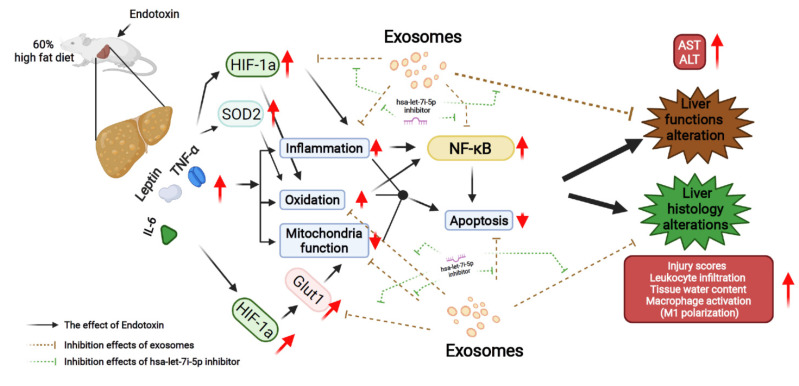
Diagram illustrating the effects and mechanisms of exosomes (from human placenta choriodecidual membrane-derived mesenchymal stem cells, pcMSCs) in inhibiting liver injury in endotoxin-treated high-fat diet obese mice, measured at 48 h after the intraperitoneal administration of endotoxin. The effects of inhibiting hsa-let-7i-5p microRNA on offsetting the effects of exosomes are also illustrated. ALT: alanine aminotransferase. AST: aspartate aminotransferase. GLUT1: glucose transporter 1. HIF-1α: hypoxia-inducible factor-1α. IL-6: interleukin-6. NF-κB: nuclear factor-κB. SOD2: superoxide dismutase 2. TNF-α: tumor necrosis factor-α.

This study demonstrated the therapeutic potential of exosomes from human placenta-derived MSC in preventing sepsis in obesity and the possible mechanisms involved. The proposed mechanisms are summarized in Figure 7.

It is established that exosomes are the key mediators for the therapeutic effects of MSC [14]. Notably, collective data highlighted the advantages of exosome-based therapy over MSC-based therapy. One main advantage, as aforementioned, is that exosome-based therapy provides superior therapeutic effects to MSC-based therapy [15]. The mechanisms involve mainly their differences in size. Unlike MSC that are too large to circulate and cannot move beyond the first-pass capillary bed, exosomes are small and thus can circulate readily [15]. Exosomes therefore can achieve a higher dose in tissues than MSC [15]. The mechanisms also involve differences in their capacity for reprogramming. Unlike MSC, exosomes lack elaborate metabolic activities and thus are unlikely to be reprogrammed [15]. Therefore, exosomes possess more definite biological and functional activities than MSC [15]. Collective data also highlighted several other advantages of exosome-based therapy over MSC-based therapy. For instance, though MSC-based therapy is essentially safe, previous data did indicate its potential in prompting endogenous tumor formation [28]. In contrast to cellular products, exosomes cannot self-replicate and thus exosome-based therapy lacks the potential of prompting endogenous tumor formation [28]. In addition, high clean room standards are required to avoid biological contamination of MSC [29]. Exosomes, in contrast, can be sterilized by filtration [29]. This advantage significantly reduces the risk of biological contamination of exosome-based therapy, compared to MSC-based therapy. As exosomes are much easier to handle than MSC, this advantage allows exosome-based therapy to be produced in a scaled manner, compared to MSC-based therapy [29]. Exosomes-based therapy can therefore serve as an attractive alternative for MSC-based therapy. Moreover, the potent therapeutic capacity and the abovementioned advantages further highlight the clinical feasibility of exosome-based therapy and support its future use in standard therapy. Exosome-based therapy therefore has great potential and warrants further investigation.

Nevertheless, our study has several limitations. First, whether exosomes from human placenta-derived MSC can produce similar preventive effects against sepsis induced by different models of sepsis (e.g., polymicrobial sepsis) [30] remains unclear. Second, the long-term effects of exosomes from human placenta-derived MSC with single or multiple boluses remain to be elucidated. Third, the surviving mice in the HFDLPS and HLEi groups were extremely weak and could not tolerate an insulin resistance test. Therefore, whether exosomes from human placenta-derived MSC can mitigate insulin resistance in obesity with sepsis remains to be determined. Fourth, the role of other miRNAs (e.g., miR-612) or other components (e.g., proteins, DNA, mRNA, long noncoding RNA, and circular RNA) [31] in exosomes from human placenta-derived MSC in preventing sepsis in obesity remains unclear. Fifth, the role of adiponectin (the anti-inflammatory protein secreted by adipocytes) in preventing sepsis in obesity [32] was not noted to be significant in this study; however, further study is required before conclusions can be drawn on this subject. Sixth, whether exosomes from other MSCs can produce similar effects in preventing sepsis in obesity requires further study.

## 4. Materials and Methods

### 4.1. Isolation, Purification, and Characterization of Exosomes

Human placenta choriodecidual membrane-derived MSCs (pcMSCs) were isolated and expanded [33]. Exosomes from human pcMSCs were then isolated through culture medium harvesting, centrifugation, supernatant collection, filtration, and ultracentrifugation (Beckman Coulter Optima L-80XP Ultracentrifuge; 100,000× *g*; 4 °C; 90 min; Type 50.2 Ti rotor, k-factor: 157.7) [34]. The resulting pellets were resuspended, pooled, ultracentrifuged, resuspended, purified, and ultracentrifuged again. The top fractions of the gradient were collected, diluted, and centrifuged. The pellets were resuspended and stored at −80 °C. Exosome particles and their size were analyzed (NanoSight NS300; Malvern Panalytical, Malvern, UK) [35], and their morphology was evaluated using transmission electron microscopy (TEM) [36] and markers for CD63 and CD9 were detected using an immunoblotting assay [36]. Details of the TEM and immunoblotting assays are provided in subsequent sections of this study.

### 4.2. Analysis of the miRNA in Exosomes

After being isolated from the exosomes and their quantity and quality determined (Invitrogen Qubit microRNA assay kit, QubitmicroRNA reagent kit; both from Thermo Fischer Scientific, Waltham, MA, USA), the miRNAs were ligated and hybridized to probes [37]. The hybridized probes were purified and counted (nCounter Prep Station and Digital Analyzer; NanoString Technologies; Seattle, WA, USA). The miRNAs were assayed (Human NanoString nCounter miRNA microarray assay), compared (nCounter Human miRNA Panel v2), and analyzed (nSolver 2.0 software; all from NanoString). The obtained miRNA data are available on the Gene Expression Omnibus database (accession number GSE97123).

### 4.3. Electroporation of the miRNA Inhibitor into Exosomes

Hsa-let-7i-5p was the most abundant miRNA identified in exosomes (Figure 1D). The role of let-7i-5p miRNA was investigated by employing a let-7i-5p miRNA inhibitor (Biotools, New Taipei City, Taiwan). The sequence used for let-7i-5p inhibition was 5′-AACAGCACAAACUACUACCUCA-3′. Electroporation of the miRNA inhibitor into exosomes was performed through precipitation, resuspension, transferal, electroporation (150 V/100 μF), removal of free-floating miRNA, and ultracentrifugation [38]. The pellet was resuspended and stored at −80 °C.

### 4.4. High-Fat Diet-Induced Obesity and Endotoxin-Induced Monomicrobial Sepsis Models

Adult male C57BL/6J mice (age: 7–8 weeks; National Laboratory Animal Center, Taipei, Taiwan) were maintained with a 12-h light–dark cycle; they were provided with a standard laboratory mouse diet and free access to water, in accordance with the care and handling guidelines of the National Institutes of Health (NIH), USA. After acclimation, mice were fed a high-fat diet (HFD: 60% kcal from fat, 20% kcal from carbohydrates, and 20% kcal from protein; Research Diets, New Brunswick, NJ, USA) for 12 weeks to induce obesity [39]. Intraperitoneal (ip) administration of gram-negative endotoxin (*Escherichia coli* 0127:B8; Sigma-Aldrich, Burlington, MA, USA) was performed to induce endotoxemia and liver injury [26]. The HFD mice were randomly allocated to receive normal saline (NS, 0.5 mL, ip), endotoxin (10 mg/kg, ip), endotoxin with exosomes (1 × 10^8^ particles per mouse, ip), or endotoxin with miRNA inhibitor-pretreated exosomes (1 × 10^8^ particles per mouse, ip), denoted as the HFD, HFDLPS, HLE, and HLEi groups, respectively. Exosomes or miRNA inhibitor-pretreated exosomes were administered at 2 h after endotoxin or NS administration.

### 4.5. Biodistribution of Exosomes Using an Ex Vivo Bioluminescence Imaging Assay

Exosomes (1 × 10^8^ particles per mouse, ip) were conjugated with Cy7 mono NHS ester (Amersham Biosciences, Buckinghamshire, UK) and injected into a set of endotoxin-treated HFD mice. Mice were euthanized at 0, 2, or 24 h after exosome administration and their organs were collected for bioluminescence imaging assay and analysis (IVIS Lumina XRMS and Living Image software; both from PerkinElmer, Waltham, MA, USA) [27].

### 4.6. Survivorship, Blood Sampling, Liver Harvesting, and Wet/Dry Weight Ratio

Mice were monitored for 48 h after endotoxin or NS administration. Surviving mice were anesthetized (zoletil/xylazine, 40/10 mg/kg, ip) and received a midline laparotomy. Blood samples were collected (through aortic puncture) and centrifuged, and the plasma was collected and stored at −20 °C. Euthanasia by decapitation was then performed. The right and median lobes of the liver were collected and immediately frozen with liquid nitrogen and were stored at −80 °C. The left lobe of the liver was collected and placed in 10% formalin (Sigma-Aldrich) for histological analysis [39]. The caudate lobe of the liver was collected, weighed, heated (80 °C, 24 h), and weighed again. The wet/dry (W/D) ratio was calculated to determine the water content of the liver [27].

### 4.7. Liver Enzyme and Metabolic Profile

Plasma concentrations of ALT and AST were measured (Vitros 750 autoanalyzer; Johnson & Johnson, New Brunswick, NJ, USA) to determine the presence of liver injury [27]. Plasma concentrations of glucose, total cholesterol, and triglyceride were measured (Roche Hitachi 917 Chemistry Analyzer; International Diagnostic Equipment, LLC, Temecula, CA, USA) to determine metabolic profiles [40].

### 4.8. Histological Analysis of the Liver

Formalin-fixed liver tissues were placed in paraffin wax, sectioned, and stained with hematoxylin and eosin. Histological characteristics, including steatosis (0: none; 3: severe), lobular inflammation (0: none; 3: > 4 foci), and hepatocyte ballooning (0: none; 2: many ballooned cells), were assessed and scored under a light microscope, and the sum scores were calculated to determine injury scores [40].

### 4.9. Enzyme-Linked Immunosorbent Assay

Freshly frozen liver tissues were homogenized and centrifuged, and the supernatants were collected [27]. The plasma and hepatic concentrations of TNF-α, IL-6, leptin, and adiponectin were then assayed (TNF-α, IL-6, leptin, and adiponectin enzyme-linked immunosorbent assay kits; all from Enzo Life Science, Farmingdale, NY, USA).

### 4.10. Immunohistochemistry Staining

Liver tissues (in paraffin sections) were incubated with primary antibodies against M1 phase-related protein iNOS, M2 phase-related protein CD206, or lipid peroxidation–related protein malondialdehyde (all from Abcam, Cambridge, MA, USA) [41]. All sections were observed (TissueGnostics Axio Observer Z1 microscope, TissueGnostics, Vienna, Austria) and analyzed (Image J, free software by NIH, USA; available at https://imagej.nih.gov/ij/; last accessed date: 1 August 2021).

### 4.11. Immunoblotting Assay

Proteins from freshly frozen liver tissues were extracted, segregated through electrophoresis, and transferred onto nitrocellulose membranes (Bio-Rad Laboratories, Hercules, CA, USA) [27]. Membranes were incubated with primary antibodies against CD63 (Proteintech, Rosemont, IL, USA), CD9 (Proteintech), phosphorylated-NF-κB (Cell Signaling Technology, Danvers, MA, USA), NF-κB (Cell Signaling Technology), HIF-1α (iReal Technology, Hsinchu, Taiwan), proapoptotic BAX (Abcam) [27], antiapoptotic Bcl-2 (Cell Signaling) [27], proapoptotic cleaved Caspase-3 (Cell Signaling) [27], or actin (internal standard; Sigma-Aldrich). Bound antibodies were detected through chemiluminescence (ECL Plus kit; Amersham). Band density was measured using densitometry (ImageJ).

### 4.12. Quantitative Real-Time Polymerase Chain Reaction

To assay transcriptional expression of iNOS, CD206, SOD2 (the main enzyme for clearing mitochondrial reactive oxygen species [ROS]) [42], and GLUT1 (the main transporter for glucose uptake) [43], we performed total liver RNA extraction (REzol C&T RNA Extraction Reagent; Protech Technology, Taipei, Taiwan), reverse transcription (HiScript I First Strand cDNA Synthesis Kit; Bionovas Biotechnology, Toronto, ON, Canada), and real-time polymerase chain reaction (PCR) amplification (RealQ Plus 2X Master Mix Green; Ampliqon A/S, Odense, Denmark). Primers for amplifications were iNOS forward: CAGCTGGGCTGTACAAACCTT and reverse: CATTGGAAGTGAAGCGTTTCG [44]; CD206 forward: CAGGTGTGGGCTCAGGTAGT and reverse: TGTGGTGAGCTGAAAGGTGA [42]; SOD2 forward: CAGACCTGCCTTACGACTATGG and reverse: CTCGGTGGCGTTGAGATTGTT [44]; and GLUT1 forward: GAACCTGTTGGCCTTTGTGGC and reverse: GCTGGCGGTAGGCGGGTGAGCG [43]. The mRNAs of the target genes were normalized to glyceraldehyde 3-phosphate dehydrogenase (GAPDH; internal standard) [43].

### 4.13. Hepatocyte Isolation, Treatment, and the Mitochondrial Function Assay

Primary hepatocytes were isolated from the liver tissues of the HFD mice through enzymatic digestion (Liver Dissociation Kit; Miltenyi Biotec, Bergisch Gladbach, North Rhine-Westphalia, Germany), filtration (MACS SmartStrainer; Miltenyi), washing (protein extraction buffer; Miltenyi), centrifugation, and resuspension [45]. Isolated hepatocytes were cultured (using Dulbecco’s modified eagle medium with 10% fetal bovine serum, 1 mM sodium pyruvate, 2 mM l-glutamine, and 1% penicillin/streptomycin; all from Thermo Fischer) in an incubator (37 °C with 95% humidified air and 5% CO_2_).

Hepatocytes were treated with phosphate buffered saline (100 μL), endotoxin (100 ng/mL; Sigma-Aldrich), endotoxin with exosomes (1 × 10^9^ particles), or endotoxin with miRNA inhibitor-pretreated exosomes (1 × 10^9^ particles). The endotoxin dose was determined according to our previous report [46]. After 48 h, the mitochondrial function of the hepatocytes was determined through a respiratory function assay (Seahorse XF Analyzer; Agilent Technologies, Santa Clara, CA, USA), which involved recording the oxygen consumption rate (OCR) in the basal condition and after mitochondrial respiration modulators were added; ATP production and the maximum OCR were also determined [47].

### 4.14. TEM Analysis

To analyze the morphology of the exosomes using TEM, an exosome suspension was fixed, transferred to grids (Polysciences, Warrington, PA, USA), dried, and observed (Hitachi HT-7700; Hitachi, Tokyo, Japan) [36]. Through a similar procedure, mitochondrial injury in isolated hepatocytes was also analyzed using TEM [48]. Six grids from each group and four random fields per grid (2 μm^2^) were selected for analysis. Alterations in mitochondrial ultrastructure were assessed using a scoring system, where 0, 1, 2, 3, 4, and 5 indicated normal appearance, minimal mitochondrial swelling, mild mitochondrial swelling, moderate or focal high-amplitude swelling, diffuse high-amplitude swelling and disruption of crystal membrane integrity, and high-amplitude swelling with some mitochondrial flocculent densities and calcifications, respectively [48]. Sum scores were calculated to determine the mitochondrial injury scores.

### 4.15. TUNEL Assay

DNA fragmentation in liver tissues was examined using the TUNEL assay (in situ cell death detection kit; Roche, Indianapolis, IN, USA). Apoptotic cells and their nuclei were stained with 4′,6-diamidino-2-phenylindole (Sigma-Aldrich). After the cells were scanned, five random fields (0.25 mm^2^) were selected for calculating TUNEL-positive cells [27].

### 4.16. Statistical Analysis

Data were calculated to determine the mean ± standard deviation. Between-group differences were analyzed using a one-way analysis of variance with a Tukey’s test for post hoc analysis. The 48-h survival rates were analyzed using the Kaplan–Meier analysis. A *p* value of <0.05 was considered significant. SPSS v21.0 (SPSS, Somers, NY, USA) was employed.

## 5. Conclusions

Data from the present study demonstrate that exosomes from human placenta-derived MSC mitigate endotoxin-induced mortality and liver injury in obese mice, and the effects can be partly mediated by let-7i-5p miRNA.

## Data Availability

All data are reported in the present study.
